# The role of environmental sensitivity in the development of rumination and depressive symptoms in childhood: a longitudinal study

**DOI:** 10.1007/s00787-021-01830-6

**Published:** 2021-06-25

**Authors:** Francesca Lionetti, Daniel N. Klein, Massimiliano Pastore, Elaine N. Aron, Arthur Aron, Michael Pluess

**Affiliations:** 1 Department of Neuroscience, Imaging and Clinical Science, G. d’Annunzio University of Chieti-Pescara, Chieti, Italy; 2 Department of Biological and Experimental Psychology, Queen Mary University of London, London, UK; 3 Department of Psychology, Stony Brook University, Stony Brook, USA; 4 Department of Developmental Psychology and Socialization, University of Padova, Padova, Italy

**Keywords:** Depression, Rumination, Internalizing problems, Environmental sensitivity, Parenting

## Abstract

Some children are more affected than others by their upbringing due to their increased sensitivity to the environment. More sensitive children are at heightened risk for the development of internalizing problems, particularly when experiencing unsupportive parenting. However, little is known about how the interplay between children’s sensitivity and parenting leads to higher levels of depressive symptoms. In the current study, we investigated the interaction between early parenting and children’s sensitivity on levels of depressive symptomatology in middle childhood, exploring the role of rumination as a possible mediator in a community sample. Participants included 196 USA resident families, from a middle class and mostly European–American background, and their healthy children, followed up from age 3 until 9 and 12 years. Environmental sensitivity was assessed observationally when children were 3 years old. Parenting style was based on parent-report at the age of 3 years. When children were nine, they completed questionnaires on rumination and depressive symptoms (repeated at 12 years). Analyses were run applying a Bayesian approach. Children’s sensitivity interacted with permissive parenting in predicting rumination at age 9. Rumination, in turn, was associated with depressive symptoms at age 9 and, to a lesser extent, at age 12. No relevant interactions emerged for authoritative and authoritarian parenting. Sensitive children may be at heightened risk for internalizing problems when exposed to a permissive parenting style. Permissive parenting was associated with increased ruminative coping strategies in sensitive children which, in turn, predicted higher levels of depression. Hence, rumination emerged as an important cognitive risk factor for the development of depressive symptoms in sensitive children.

**Supplementary Information:**

The online version contains supplementary material available at 10.1007/s00787-021-01830-6.

## Introduction

Internalizing problems in youth are common and have likely increased over the last decade, as documented in reviews and epidemiological studies [1–3]. They tend to emerge in childhood and are relatively stable throughout life [4, 5]. Hence, a better understanding of what contributes to the development of such problems in childhood is crucial. Among environmental factors, a variety of early stressors related to the parent–child relationship and to parental behaviours, beliefs and attitudes, including insecure parent–child attachment relationships, parental over-involvement, endorsement of harsh parenting practices, over-reactive parenting, authoritarian parenting, and permissive parenting have been reported as predictors of internalizing problems in children [4, 6, 7]. However, a growing number of studies provide evidence that child characteristics such as temperament traits also contribute to the aetiology of internalizing disorders through both additive and moderating effects [8–10].

One important child characteristic that might be of importance for the study of internalizing symptoms is environmental sensitivity (ES), a trait capturing constitutionally based individual differences in sensitivity to environmental influences [11]. Environmental sensitivity has been defined as the ability to perceive and process information about the environment and can be measured reliably per questionnaire [12, 13] or behavioural observation [14]. Deeper processing paired with higher emotional reactivity is deemed to be the core component of heightened ES, which is manifested behaviourally in higher inhibition when approaching new environments to allow time to process stimuli [14–16]. According to empirical studies, a significant minority of individuals, about 20–30%, score particularly high on this trait [8, 12, 17], and are more susceptible to the quality of their environment, whether negative or positive [8, 13]. High ES has been found to correlate with internalizing symptoms such as anxiety and depression in adult samples [18] as well as excessive crying, medically unexplained physical symptoms, and sleeping, eating, and drinking problems in children [19]. Similarly, meta-analytic data reported significant correlations with negative affect and neuroticism*,* with effect sizes of *r* = 0.40 and *r* = 0.34, respectively, in adult samples, and of *r* = 0.29 for negative affect in children [20]. Importantly, findings in adult samples suggest that it is the interaction between ES and environmental quality, rather than their individual effects, that predict internalizing symptoms [18, 21]. Regarding children, a recent study provided evidence that sensitive children had the highest levels of internalizing behavioural problems at ages 3 and 6 when they experienced permissive parenting in early childhood [14]. However, when permissive parenting was low, these children were no more at risk than their low-sensitive peers. Similar findings have been reported in another study involving pre-schoolers, with internalizing problems at age 4 found to be greatest among behaviourally inhibited children exposed to high levels of permissive parenting [22]. Behavioural inhibition has been reported as one of the key features of individuals high in ES when approaching new environments [14, 15], to allow an in-depth processing of stimuli [23]. Hence, the results reviewed above suggest that a parenting style that does not involve establishing clear boundaries and providing directions might be a specific risk factor for higher levels of negative thoughts in children who tend to be more cautious and reactive to novelty, and process information more deeply.

Taken together, the scientific literature converges on the notion that highly sensitive children may be at increased risk for internalizing problems, with some studies suggesting that this is especially true in less than optimal environments. However, what remains largely unexplored is the identification of mechanisms that explain how heightened sensitivity may increase the risk for the development of internalizing symptoms. The hypothesis that we propose here is that the tendency of highly sensitive children to process information more deeply [13–16] can lead to negative cognitive patterns, but only in less supportive developmental contexts. Depth of processing, characterized by deeper cognitive processing of stimuli as captured via questionnaires, and greater activation in secondary perceptual processing brain areas as shown in fMRI studies [24, 25], might lead to a more ruminative thinking style in children high in ES if the surrounding environment has not been able to promote the development of positive strategies for dealing with negative thoughts. Rumination, that is the tendency to repeatedly reflect on the same negative thoughts [26, 27], identified as one key mechanism in the onset of internalizing symptoms, and particularly depression [28–30], might represent an important mechanism for the higher levels of internalizing problems in highly sensitive individuals. However, as far as we are aware, the role of rumination in the development of higher levels of depressive symptoms in sensitive children has not yet been investigated; furthermore, the existing literature investigating sensitivity and internalizing symptoms is characterized by several methodological limitations. First, the majority of studies rely on a single informant, use recalled retrospective parenting quality, and apply self-reported sensitivity (for review, see [16]). Second, despite a number of studies investigating associations between ES and internalizing symptoms, very few have tested the interplay between ES and childhood environment in the prediction of internalizing symptoms [18, 21]. Third, most existing studies did not explore the mechanisms that may explain associations between ES and higher levels of depressive symptoms.

Overcoming some of these limitations, the current study aims to investigate the interaction between early parenting styles and children’s ES on rumination and depressive symptoms in middle childhood and early adolescence. The study features the same sample of U.S. children and their mothers from the Stony Brook temperament study [31] who were included in a recent study reporting significant interactions between observed ES at age 3 and permissive parenting in the prediction of internalizing behavioural problems at age 3 and 6 years [14]. Specifically, we aimed to investigate the interaction between early parenting style and ES on internalizing symptoms when children were 9 and 12 years old (which was not available in the original study). We hypothesized a stronger association between parenting at age 3 and internalizing problems in middle childhood for sensitive children and that, given the depth processing characterizing children scoring high in ES, rumination would be the mediating process. Informed by previous findings on the impact of parenting on internalizing symptoms [14], we further hypothesized that sensitive children’s internalizing symptoms would be especially strongly predicted by permissive rather than authoritarian and authoritative parenting styles. An authoritarian parenting style tends to have adverse and an authoritative parenting style beneficial effects for most children. However, highly sensitive children may be especially vulnerable to the negative effects of a permissive parenting style, characterized by the absence of structure and guidance. Due to the tendency of sensitive children to process information more deeply, the lack of structure and positive disciplinary strategies may result in difficulties controlling the processing of negative thoughts and feelings [14]. To integrate our hypotheses and previous findings when children were aged 3 and 6 years, with the current data at ages 9 and 12 years, we adopted a fully Bayesian approach with informative priors for data analysis. This allowed us to incorporate previous knowledge into the analysis of new data, and to more efficiently estimate the magnitude of expected effects rather than testing the null hypothesis. As further detailed below, implementing Markov chains Monte Carlo (MCMC) sampling increased the robustness of estimated parameters even with a relatively small sample size [32]. Parameters estimated with the traditional maximum likelihood approach and related *p* values are reported in the supplementary material file (Sect. 4). Results converged on the same conclusions.

## Methods

### Participants

Participants were drawn from the stony brook temperament study (SBTS), an ongoing longitudinal study in the USA [31], involving children from the community, recruited through commercially obtained mailing lists, with no significant medical conditions or developmental disabilities, and living with at least one English speaking biological parent. Participants were primarily European–American (87%), came from two-parent homes (94%) and had a middle-class background [33].

All procedures contributing to this work comply with the ethical standards of the relevant national and institutional committees on human experimentation and with the Helsinki Declaration of 1975, as revised in 2008*.* Institutional Review Board approval for the current study was obtained from Stony Brook University (study name: Observations of Active and Inactive Children*,* protocol number: 88933–35). In the current study, we considered a subset of children from the Stony Brook temperament study for whom data on both parenting and observed ES were available at age 3, and who were included in our previous analysis on the interaction between early parenting and children’s ES in the prediction of behavioural problems at ages 3 and 6 [14]. For the purpose of this study, we included additional data at ages 9 and 12. At age 3, data were available for 292 children (see [14]), at age 9, due to attrition over time, data were available for 214 children, and at age 12 the sample included 196 children (43% female). Importantly, the sample with complete data (*n* = 196) was comparable to the original sample across all study variables (*N* = 292, see supplementary material file, Sect. 1).

### Procedure

When children were 3 years old, mothers provided information on parenting, and children’s ES was observed in a laboratory context. At 9 years, children provided information on ruminative coping strategies and at both 9 and 12 years on depressive symptoms.

### Measures

Environmental sensitivity. Children’s sensitivity was investigated at age 3 with the highly sensitive child-rating system (HSC-RS) [14]. The HSC-RS consists of a set of 10 rating scales that code global behaviours associated with sensitivity observed in the context of the Lab-TAB procedure [34]. Each scale ranges from 1 to 7, with higher scores reflecting higher levels of ES. Psychometric proprieties of the HSC-RS were satisfactory [14].

Parenting. Parenting style was assessed when children were aged 3 using the parenting styles and dimensions questionnaire (PSDQ) [35]. Parents reported on three parenting styles: permissive (five items capturing an indulgent caring attitude with difficulties in setting rules), authoritarian (12 items capturing low emotional support and hostility), and authoritative parenting (15 items capturing emotional support and rule setting reasoning). Internal consistency based on the 414 mothers who completed the questionnaire when children were 3 years old was good with Cronbach’s *α* = 0.74, 95% CI (0.70–0.77) for permissive parenting, *α* = 0.74, 95% CI (0.70–0.77) for authoritarian parenting, and *α* = 0.82, 95% CI (0.80–0.84) for authoritative parenting.

Rumination. At age 9, children completed the ruminative response subscale from the child response styles questionnaire (CRSQ-Rumination) [36]. Children were instructed to select the option that indicates how they usually respond to feeling sad on a four-point scale. Internal consistency based on data from the 425 children who completed the questionnaire in the entire SBTS sample was good with *α* = 0.84, 95% CI (0.82–0.86).

Depressive symptoms. At 9 and 12 years, children reported levels of depressive symptoms using the children’s depression inventory (CDI) [37]. Children were instructed to select the response for each item that best describes how they were thinking and feeling during the past week. Items are scored on a third-point scale, with higher scores reflecting greater depressive symptoms. Internal consistency, based on the entire SBTS sample, was acceptable with Cronbach’s *α* = 0.74, 95% CI (0.71–0.77), at age 9 (*N* = 481) and *α* = 0.82, 95% CI (0.79–0.84) at age 12 (*N* = 357).

### Data analysis

**Fig. 1:**
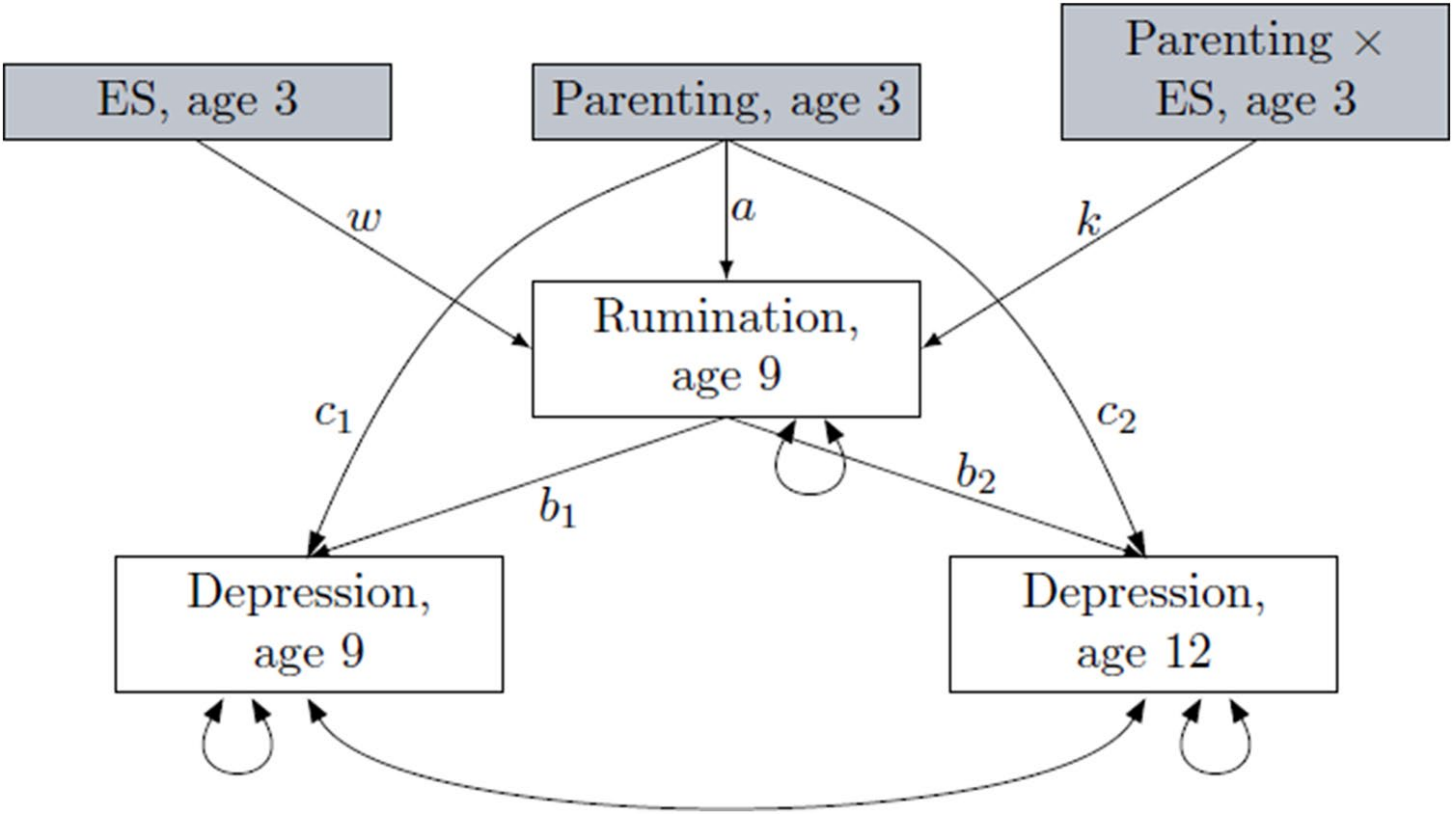
Graphic illustration of model 3: parenting at age 3 predicting depression at ages 9 and 12 directly and through the mediating role of rumination at age 9, conditional on environmental sensitivity (ES) assessed at age 3. Double arrows represent variances and covariances of exogenous variables (dotted lines) and of residual errors (straight lines). In model 2, path *k* was not included*,* in model 1 both paths *k* and *w* were not included

Analyses were performed using the statistical software R [38], including blavaan [39] using STAN for implementing Markov chains Monte Carlo (MCMC) sampling [40, 41] and ggplot2 [42] packages. First, we computed bivariate correlations among study variables using Pearson’s *r*. Afterwards, we compared and explored a series of multivariate regression models adopting a Bayesian approach for estimating parameters. The specific models that we compared were: model 0, the null model, i.e. a model assuming that there is no correlation among study variables, model 1, representing the association of parenting at age 3 with depression at 9 and 12, mediated through rumination at age 9, model 2 which was similar to model 1 but included the additive effect of ES on rumination at age 9, and model 3 which added the interaction term between parenting and sensitivity on rumination to test whether parenting predicted depression through the mediating role of rumination, conditional on ES levels (see Fig. 1). Models 0–3 were repeated separately for each of the three parenting styles. Depression at ages 9 and 12 was simultaneously included in all tested models. The following comparative indices were used to compare models: leave-one-out cross-validation information criterion (Loo IC) [43], with lower values reflecting a better fit of the model to data, the log Bayes factor, specific to the comparison with the null model, with higher values providing a stronger support to the model with respect to the null one, and the model weight criterion [44], with higher values reflecting a stronger support for the model. Loo IC and the log Bayes factor were used to compare each model against the null one, and the model weight criterion to compare each model against the previously tested model. In addition, for each endogenous variable, the variance explained by predictors was explored using *R*^2^ associated 90% highest posterior density intervals (HPDI) [45, 46] on the best model selected. HPDI values provide a direct representation of the most credible values of estimated parameters after accounting for prior beliefs. If HPDIs do not contain the zero, or only a small proportion of values are close to zero, then an effect can be reasonably supported.

Prior distributions*.* We defined informative prior distributions to incorporate our expectations (defined by prior mean value) and associated uncertainty (defined by prior standard deviation) into the analysis of the new data. We used standardized prior distributions, priors were defined based on results from the same sample when participants were aged 3 and 6 years [14]. We expected the pattern of findings that we previously identified in relation to internalizing behavioural problems to be stable at ages 9 and 12, that is, to identify an association between permissive parenting and internalizing symptoms specific for children scoring high in ES. At the same time, we assumed a moderate degree of uncertainty to consider the possibility of different findings. More specifically, pertaining to the impact of parenting at age 3 on rumination at age 9 (path *a,* Fig. 1), we considered data from the same sample [14] showing that parenting had a small impact on behavioural and social outcomes at age 6, whilst also considering literature reporting that parenting styles matter for children’s adjustment [6]. Hence, we hypothesized a relatively small but noticeable impact, with a moderate degree of uncertainty, operationalized as a normal distribution with a mean of *M* = 0.1 and a standard deviation of *SD* = 0.1, formally written as normal (0.1, 0.1), with a positive direction of effects of permissive and authoritarian parenting, and a negative direction for authoritative parenting. Similarly, pertaining to parenting predicting depression at ages 9 (path *c*1) and 12 (path *c*2), we expected a small but noticeable relation, equal to normal (0.1, 0.1) and normal (0.05, 0.1) for ages 9 and 12, respectively. Regarding predicting the impact of rumination at age 9 on depression at age 9 (path *b*1), considering the literature showing that the two are strongly associated [27–29], we hypothesized a relatively strong association with normal (0.50, 0.10). For rumination at age 9 on depression at age 12 (path *b*2, Fig. 1), we expected a smaller effect size compared to that observed for the two variables at age 9, with normal (0.35, 0.10). For the association between ES at age 3 and rumination at age 9 (path *w*), we adopted a sceptical prior with normal (0, 0.2), as we did not expect sensitivity at age 3 to predict rumination at age 9 irrespective of environmental quality, but we still allowed the possibility of an association between the two. For the interaction term between parenting and ES (path *k*, Fig. 1), based on previous empirical literature [14, 22], we assumed that permissive parenting would be a specific risk factor for highly sensitive children, hence we operationalized the interaction between permissive parenting and ES with normal (0.3, 0.1), and we assumed no significant interaction between ES and authoritative and authoritarian parenting [normal (0, 1)]. Given that residual variances are positive by definition, we used the default prior distribution for residual variances in the blavaan package for [39], namely a gamma distribution formally written as gamma (1, 0.5).

Computational details. Posterior distributions for each parameter were estimated using four Markov chains Monte Carlo (MCMC), each running at least for 4000 replicates. MCMC convergence was assessed by calculating the potential scale reduction statistic, PSRF. This statistic measures the ratio of the average variance of samples within each chain to the variance of the pooled samples across chains.

Interpretation of posterior distributions. Once the best model was identified, we considered standardized posterior distributions of model parameters to interpret effects. Each posterior was summarized by its mean value and associated 90% highest posterior density intervals, as described above. In addition to this, following Kruschke and Liddell [47], we evaluated effects considering the region of practical equivalence (ROPE), which defines values that are equivalent to the null effect. The lower the percentage of overlap between the ROPE and HPDI, the stronger is the support provided for the investigated effect. To summarize effects, we used the inverse of the overlap computed with *I* = 1− (HPDI ∩ ROPE)/HPDI), with *I* varying from 0.0 to 1, so that higher values corresponded to stronger effects. The ROPE was set from −0.1 to + 0.1 for all model parameters representing direct effects [47]. For indirect effects, as these are function of investigated parameters (*a*×*b*1 and *a* × *b*2 for indirect effects, and *a* × *b*1 + *k* × *b*1 and *a* × *b*2 + *k* × *b*2 for conditional indirect effects), ROPEs were (−0.01, 0.01) and (−0.02, 0.02), respectively, for indirect and conditional indirect effects.

Model predictions and interaction effects. Finally, we illustrated interaction effects and findings for extreme groups [i.e. highly sensitive children (scoring in the top 30%) and low-sensitive children (scoring in the bottom 30%)] [12].

To ensure that findings were not biased by missing data, we repeated all analyses in an imputed data set, applying a Bayesian estimation method described in the supplementary material (Sect. 3). Given that the imputed results were very similar to non-imputed data, we decided to only report results based on the available data (we provide results from the imputation and the associated sensitivity analysis in supplementary materials).

## Results

### Descriptive statistics

Univariate statistics and bivariate correlations (*N* = 196) are reported in Table 1. ES (measured behaviourally at year 3) correlated positively with female gender (*r* = 0.19), and negatively with depression at age 9 (*r* = −0.21). The correlation between ES and rumination at age 9 was positive and small (*r* = 0.10). Rumination correlated positively with depression at age 9 (*r* = 0.35) and, to a lesser extent, with rumination at age 12 (*r* = 0.14). Authoritarian parenting (assessed at age 3) was positively associated with depression at age 9 (*r* = 0.14), whereas authoritative parenting was negatively associated with depression at age 9 (*r* = 0.15). Authoritative parenting was negatively associated with rumination (*r* = −0.14). Negligible associations were identified between permissive parenting and internalizing symptoms at both ages. Associations between the age 3 parenting variables and depression symptoms at age 12 were trivial.

**Table 1:** Univariate statistics of study variables and bivariate correlations (*N* = 196)

	Mean (SD)	1	2	3	4	5	6	7
Environmental sensitivity (age 3)	4.00 (0.90)	–						
Sex (0 = male, 1 = female)	0.43	0.19	–					
Permissive parenting (age 3)	10.75 (3.04)	0.01	0.05	–				
Authoritarian parenting (age 3)	20.08 (4.18)	−0.07	−0.05	0.36	–			
Authoritative parenting (age 3)	60.69 (6.65)	−0.07	0.08	−0.08	−0.13	–		
Rumination (age 9)	23.41 (7.02)	0.10	0.00	0.07	0.08	−0.14	–	
Depression (age 9)	4.53 (3.76)	−0.21	−0.18	0.07	0.14	−0.15	0.35	–
Depression (age 12)	4.55 (5.39)	−0.01	0.16	−0.03	0.07	0.01	0.14	0.33

### Multivariate regression models

Results of model comparisons, used to identify the best fitting model, are reported in Table 2.

**Table 2:** Comparison of multivariate regression models

	Permissive parenting				Authoritative parenting				Authoritarian parenting			
	Looic	LogBF	*w*	*w*_1_	Looic	LogBF	*w*	*w*_1_	Looic	LogBF	*w*	*w*_1_
Model 0	3369	0	0		3354	0	0		3329	0	0	
Model 1	1637	872	0.03	> 1000	**1634**	**866**	**0.42**	**> 1000**	1635	854	0.46	> 1000
Model 2	1637	49	0.03	1	1634	866	0.41	0.98	**1635**	**854**	**0.47**	**1.02**
Model 3	**1630**	**874**	**0.94**	31.33	1636	864	0.17	0.41	1639	850	0.08	0.17

Model 0 is the null model, model 1 is the model with parenting predicting depression through the mediating role of rumination, model 2 includes the additive role of environmental sensitivity on children’s rumination, and model 3 the interaction term between environmental sensitivity and parenting on rumination, as depicted in Fig. 1. The best fitting model for each parenting style is marked in bold. All are estimated against the null model. *w*_*1*_ is model weight against the previously tested model. Models are estimated on *N* = 196 subjects

*Looic* leave-one-out cross-validation information criterion, *logBF* log-Bayes factor, *w* model weight.

**Fig. 2:**
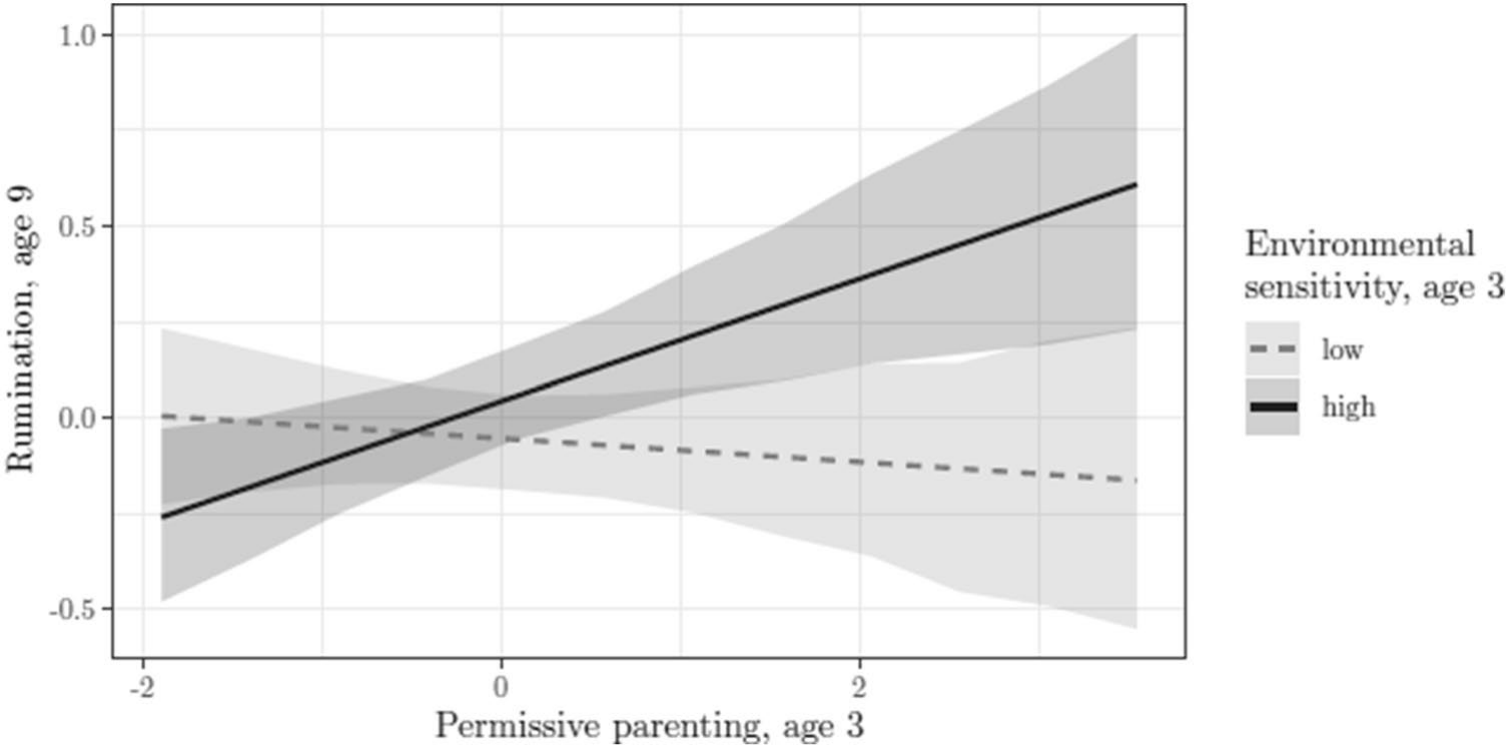
Model predictions. Values of rumination at age 9 as a function of permissive parenting at age 3, in low (bottom 30% of the HSC distribution)- and high (upper 30% of the HSC distribution)-sensitive children. Band lines represent 90% HPD intervals. Values are −0.03 for low-sensitive children, and 0.16 for high-sensitive children

Permissive parenting. Comparative indices provided support for model 3, with permissive parenting interacting with ES in the prediction of rumination at age 9 which, in turn, was associated with higher levels of depression at both age 9 and 12. Credible intervals of the explained variance for each exogenous variable did not contain the zero for the best model selected, with *R*^2^ 90% HPDI (0.03, 0.12) for rumination at age 9, *R*^2^ 90% HPDI (0.11, 0.22) for depression at age 9, and *R*^2^ 90% HPDI (0.01, 0.09) for depression at age 12, suggesting that the effects can be reasonably supported with stronger effects at age 9 compared to age 12. More specifically, posterior distributions and associated 90% HPDI showed that permissive parenting did not have a direct relation to depression at age 9 and 12 [path *c*1 and *c*2 Fig. 1, respectively, *B* = 0.06, 90% HPDI (−0.02, 0.15), *I* = 0.28, and *B* = −0.007, 90% HPDI (−0.09, 0.86), *I* = 0.00], nor with rumination at age 9 [path *a*, Fig. 1, *B* = 0.08, 90% HPDI (−0.02, 0.17), *I* = 0.36]. Similarly, no association of sensitivity with rumination was identified [path *w*, Fig. 1, *B* = 0.11, (0.00, 0.21), *I* = 0.53]. Importantly, permissive parenting interacted with ES in predicting higher levels of rumination at age 9 [path *k*, Fig. 1, *B* = 0.23, (0.14, 0.32), *I* = 1.00]. In turn, higher levels of rumination predicted higher levels of depression at age 9 [path *b*1, Fig. 1, *B* = 0.40, (0.32, 0.49), *I* = 1] and, to a lesser extent, at age 12 [path *b*2, Fig. 1, *B* = 0.22, (0.13, 0.31), *I* = 1]. Posterior distributions also provided support for an indirect effect of permissive parenting on depression through the moderating role of rumination, conditional on ES, both at age 9 and 12 [with *B* = 0.12, (0.07, 0.18), *I* = 1 and *B* = 0.12, (0.03, 0.05), *I* = 0.1, respectively, for age 9 and 12]. Posterior distributions are depicted in the supplementary material file (Sect. 2). A graphical representation of the interaction between ES and permissive parenting on rumination at age 9 is provided in Fig. 2. Permissive parenting values are on the *X*-axis, and the model predictions of rumination are on the *Y*-axis, with regression lines representing individuals with low and high levels of ES (bottom and top 30% of observed ES). The figure shows that children scoring high on ES presented with higher levels of rumination when permissive parenting was high.

Authoritarian parenting. Model 2 received the strongest support, though the difference with model 1 was trivial. The model including the interaction between ES and parenting was not significantly better than the others. *R*^2^ 90% HPDI were (0.00, 0.04) for rumination at age 9, (0.12, 0.23) for depression at age 9 and (0.02, 0.09) for depression at age 12, suggesting that the effects can be reasonably supported on the basis that zero was not included. Similar to permissive parenting, stronger effects were found for age 9 compared to age 12. More specifically, authoritarian parenting predicted depression at age 9 [*B* = 0.10, (0.01, 0.19), *I* = 0.49] and rumination at age 9 [*B* = 0.09, (−0.00, 0.18), *I* = 0.45], but effects were overall small. Rumination predicted depression at age 9 and 12 [*B* = 0.39, (0.31, 0.48), *I* = 1, and *B* = 0.21, (0.12, 0.30), *I* = 1.00, respectively]. Consistent with the limited support that model 2 received against model 1, no relevant impact of ES on rumination was detected [*B* = 0.09, (−0.02, 0.20)]. Indirect effects at age 9 and 12 were supported [*B* = 0.03, (0.00, 0.07), *I* = 0.67 and *B* = 0.09, (−0.00, 0.044), *I* = 0.69, respectively]. Posterior distributions of all estimated parameters are reported in the supplementary materials section.

Authoritative parenting. Model 1 received the strongest support, though the difference with model 2 was trivial. The model including the interaction between ES and parenting was not significantly better than the others. *R*^2^ 90% HPDI were (0.00, 0.04) for rumination at age 9, (0.12, 0.24) for depression at age 9 and (0.01, 0.09) for depression at age 12, suggesting that the effects can be reasonably supported. Again, stronger effects were identified for age 9 compared to age 12. Posterior distributions specifically provided limited support for parenting at age 3 predicting rumination at age 9 [*B* = −0.13, (−0.22, −0.04), *I* = 0.65]. Rumination at age 9 was associated with depression at both age 9 and 12 [*B* = 0.39, (0.31, 0.47), *I* = 1, and *B* = 0.22, 90% HPDI (0.13, 0.31), *I* = 1.00. Indirect effects at age 9 and 12 were again supported and equal to *B* = −0.05, (−0.09, −0.01), *I* = 0.90, and *B* = −0.13, (−0.05, −0.00), *I* = 0.88, respectively]. Posterior distributions are reported in the supplementary materials section.

## Discussion

Several studies report significant associations between the trait of ES and internalizing symptoms in community samples, but mechanisms underpinning this link are unclear. Given that theory suggests that sensitive children tend to register and process information more deeply, one reason for the association between sensitivity and depression symptoms may be that sensitive children are more likely to dwell on negative thoughts if they have experienced a suboptimal rearing environment that is not able to support and promote the development of regulation competences. In the current study, we investigated whether highly sensitive children are more prone to ruminative strategies when the early rearing environment is less than optimal, and whether rumination in turn mediates the associations between early parenting styles and depressive symptoms when they are older, potentially explaining why highly sensitive individuals are more prone to internalizing problems than others, as frequently reported in previous studies [16, 20].

Building on results of a previous study according to which observer-rated sensitivity moderated the effects of permissive parenting at age 3 on parent-reported internalizing behavioural problems when children were 3 and 6 years old [14], and on the empirical literature suggesting that permissive parenting is a risk factor for internalizing symptoms in children who are potentially more susceptible to the influence of the environment [22], we found that the moderating effect of sensitivity measured behaviourally at age 3 extended to child-reported rumination in the same sample when children were 9 years old. Furthermore, rumination was positively associated with depressive symptom at ages 9 and, to a lesser extent, 12 years. Hence, these results suggested that early permissive parenting might represent a risk for subsequent depressive symptoms in highly sensitive children due to heightened levels of rumination in middle childhood, with stronger effects for outcomes at age 9 compared to age 12. In other words, the data suggest that sensitive children may be more at risk to develop depressive symptoms when exposed to permissive parenting because they are more likely to engage in ruminative coping strategies than less sensitive children. These findings, paired with data from other studies suggesting that sensitive children show more positive social competences [14], fewer behavioural problems [13], and more positive interactions with peers [48] when exposed to positive environments, point to the notion that highly sensitive individuals are not only more negatively affected by suboptimal environments, but also benefit disproportionately from positive ones, as postulated by other theories on the individual—environment interplay, such as the concept of differential susceptibility [8].

Concerning authoritarian parenting, results suggest that harsh disciplinary strategies increase the risk for rumination which, in turn, increases child-reported depression. However, these effects were not meaningfully moderated by sensitivity. For authoritative parenting, a somewhat similar pattern emerged with rumination mediating the protective impact of positive parenting on depression, with no moderation by sensitivity.

The finding that permissive parenting, but not authoritarian or authoritative parenting, appears to increase the risk for depressive symptoms in sensitive children is in line with previous research [14, 22] and suggests that the absence of clear boundaries and rules may make it more difficult for sensitive children to learn to regulate their thought patterns, which may increase their risk to develop higher levels of depressive symptoms. In some studies, permissive parenting has been reported to be associated with fewer adolescent depressive symptoms [49] and less stress [50]. However, it has also been found to predict unhealthier adolescent eating behaviours [50], increased risk of school drop-out [51], more internalizing and externalizing behavioural problems [52, 53], and poorer emotion regulation competences [54]. A partial reason for the unclear relationship between permissive parenting and a variety of outcomes may be that indulgent parents express affection for their children (the positive side of permissive parenting), but in some cases, this expression of affection may be excessive, possibly in an effort to eclipse other behaviours such as overreactive parenting tactics [55] or an overall lower attention to the child when it comes to confronting disciplinary actions that are part of everyday parent–child interactions [56]. Our findings shed light on this debate, and in line with previous research [22], suggest that permissive parenting is a risk factor depending on children’s individual differences in ES. Parenting a child who needs more time for approaching new environments and tends to process things more deeply may especially require parents who have the ability to provide emotional care together with age-appropriate rule-enforcement, and do not leave the child alone when confronted with new contexts and new developmental tasks, including the challenge of dealing with negative thoughts and emotions.

From a clinical and applied perspective, our findings highlight the importance of considering the temperament trait of environmental sensitivity to advance our understanding of the development of rumination and depressive symptoms in childhood, and suggest that when screening for internalizing symptoms it might be useful to include information on children’s sensitivity as well as the parenting they receive to optimize the early identification of children at heightened risk. Pertaining to intervention programs, our findings suggest that considering the specific dynamics of individual parent–child dyads can help to better understand what parenting practices are most relevant and important for specific children. In line with the classic “goodness of fit” model as proposed by Thomas and Chess [57], it is not an individual trait alone that predicts the development, rather it is the interaction between temperament and specific features of the environment which contribute to explaining the development. Hence, parents might benefit from being more aware of their children’s levels of environmental sensitivity and of the potential impact of their own affect, behaviours, as well as ways for managing rules at home, depending on their children individual differences in temperament. The role of a warm yet authoritative parenting style has been advocated by many programmes [58, 59] and might be particularly relevant for parents of highly sensitive children. In addition, results might also inform programs that directly target the child. Supporting children to develop adaptive strategies for the processing of their experiences, such as those used in cognitive behavioural therapy approaches, might be particularly helpful for the prevention and treatment of internalizing symptoms in children with heightened sensitivity, as previously shown with depression [60] and anxiety symptoms [61]. The media coverage on sensitivity has significantly increased over the last years, as well as blogs, books and non-scientific journals that discuss how to reduce the risk of psychological distress including depression and anxiety in sensitive individuals. To the best of our knowledge, this is the first study providing empirical evidence for rumination in highly sensitive children as a candidate key variable involved in internalizing problems.

This study has several strengths, including longitudinal data from age 3 to 12, and multiple methods of measurement provided by multiple informants, including parent-reported parenting style, child-reported rumination and depressive symptoms, and observer-rated sensitivity of children, in a relatively large sample for a longitudinal and observational study. Notwithstanding, findings have to be considered in light of several limitations. First, the majority of parents in our sample were US-based, white, and from a relatively low-risk population. Meta-analytic studies indeed suggest that the impact of authoritarian and permissive parenting might vary depending on the cultural context [62]. Hence, results should be considered specific to the current Western sample. Second, parenting was assessed via self-report by the parents, which might lack objectivity. Third, though the sample size was notably large given the observational assessment of ES and the longitudinal study design, even larger samples would be helpful to simultaneously explore additive and interactive effects of the various parenting styles. Finally, and important to acknowledge, results were overall modest in effect size, which may be partially explained by the long time span between assessment waves and multiple informants.

## Conclusion

Our results highlight the importance of considering both parenting quality as well as trait characteristics of children in the early identification of risk for the development of internalizing symptoms. The current study suggests that it is the combined effect of heightened sensitivity to environmental influences and permissive parenting that increases the risk for the development of internalizing symptoms rather than their main effects alone. Furthermore, our study suggests that highly sensitive children who experience higher levels of permissive parenting in their early years are more likely to develop ruminative coping strategies which are associated with higher levels of depressive symptoms. In summary, this study provides new evidence suggesting that highly sensitive children are more likely to develop a ruminative cognitive style in response to a permissive parenting style which then contributes to their heightened risk for the development of higher levels of depressive symptoms.

## Supplementary Information

Below is the link to the electronic supplementary material.

Supplementary file1 (PDF 1825 KB)

## Data Availability

Data are available upon request to the corresponding authors.

## Code availability

R code to compute Bayesian analyses is available upon request to the corresponding author.
