# Supplementary material for: The role of environmental sensitivity in the development of rumination and depressive symptoms in childhood: a longitudinal study
Source: Eur Child Adolesc Psychiatry. 2021 Jun 25;31(11):1815–25. doi: 10.1007/s00787-021-01830-6 (PMC9666332; doi:10.1007/s00787-021-01830-6)
Supplement: Supplementary file 1 — Supplementary file1 (PDF 1825 KB) [file 787_2021_1830_MOESM1_ESM.pdf]

The Role of Environmental Sensitivity in the Development of Rumination and Depressive Symptoms in Childhood: A Longitudinal Study

Supplementary materials

Francesca Lionetti[1,2], Daniel N. Klein[3], Massimiliano Pastore[4], Elaine N. Aron[3], Arthur Aron[3], Michael Pluess[2]

May 14, 2021

[1] Department of Neuroscience, Imaging and Clinical Science, G. d'Annunzio University of Chieti-Pescara, Chieti, IT
[2] Department of Biological and Experimental Psychology, Queen Mary University of London, London, UK
[3] Department of Psychology, Stony Brook University, Stony Brook, USA
[4] Department of Developmental Psychology and Socialization, University of Padova, IT

Correspondence to:
massimiliano.pastore@unipd.it
Department of Developmental Psychology and Socialization
University of Padova
Via Venezia, 8
Padova, ITALY

The current paper aimed at investigating the interaction between parenting style and children's Environmental Sensitivity on rumination and depression. A graphical representation of the target model is provided below and in the paper. The model was replicated for the three parenting styles considered, and a series of main effect and interaction models were compared to identify the best one. In particular, for permissive parenting, the model receiving most support was the one represented in Figure 1. For authoritarian parenting, the model receiving most support was the model excluding the interaction term between parenting and Environmental Sensitivity (the k parameter in Figure 1). For authoritative parenting, the model receiving most support was the one excluding the variable Environmental Sensitivity (ES) and consequently also the interaction between ES and parenting (the w and k parameters in Figure 1).

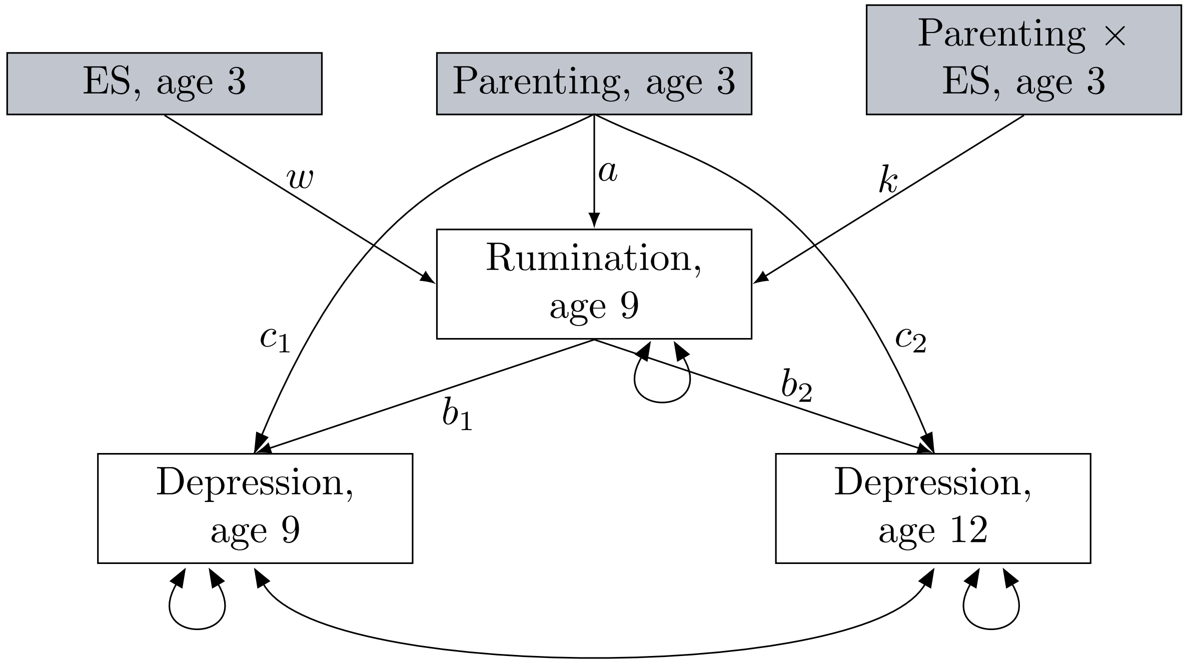

Figure 1: Target model.

1 Exploration of differences between the original sample (Lionetti, Aron, Aron, Klein, & Pluess, 2019) and the current sample

Due to attrition over time, the sample at age 9 and 12 ($n = 196$) included less subjects than the sample at age 3 and 6 (Lionetti et al., 2019). To explore if the two groups differ in regard to model variables, we compared score distributions using empirical densities (see Figure 2) and cumulative distribution (see Fig. 3).

For quantifying the degree of similarity we computed the overlapping index η (reported in Table 1, Pastore & Calcagnì, 2019) representing the proportion of overlapping between pairwise density distributions. The overlapping index η ranges from 0 (when distributions are completely disjoint) to 1 (when are completely overlapped). Both graphical representations and η values suggested that there were no relevant differences beteween samples.

	η	A	B
Permissive_Parenting_age3	0.83	64	196
Authoritarian_Parenting_age3	0.85	64	196
Authoritative_Parenting_age3	0.71	64	196
Rumination_age9	0.85	44	196
Depression_age9	0.83	44	196
Depression_age12	0.88	37	196
ES	0.79	92	196

Table 1: η is the proportion of overlapping between empirical densities represented in Figure 2. n_A and n_B are frequencies; A refers to subjects with missing data, B to the sample used in the paper.

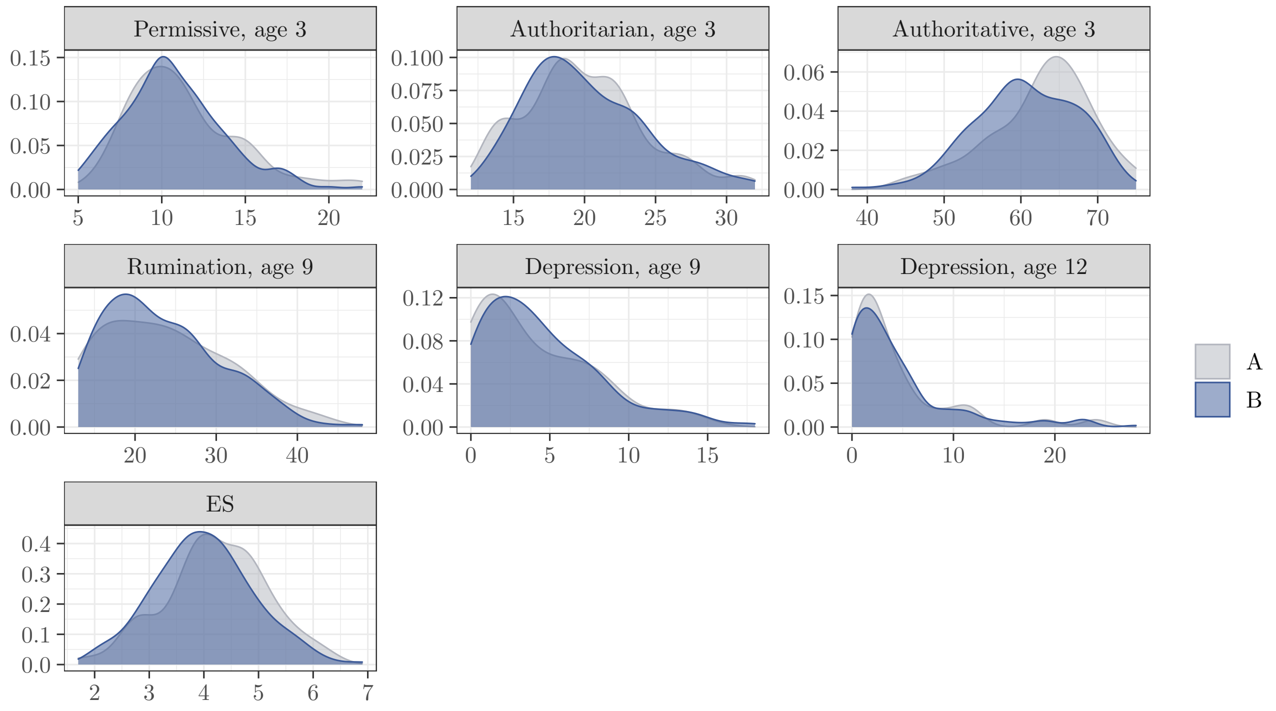

Figure 2: Empirical density of model variables depending on the presence of missing data. A refers to subjects with missing data, B to the sample used in the paper ($n = 196$)

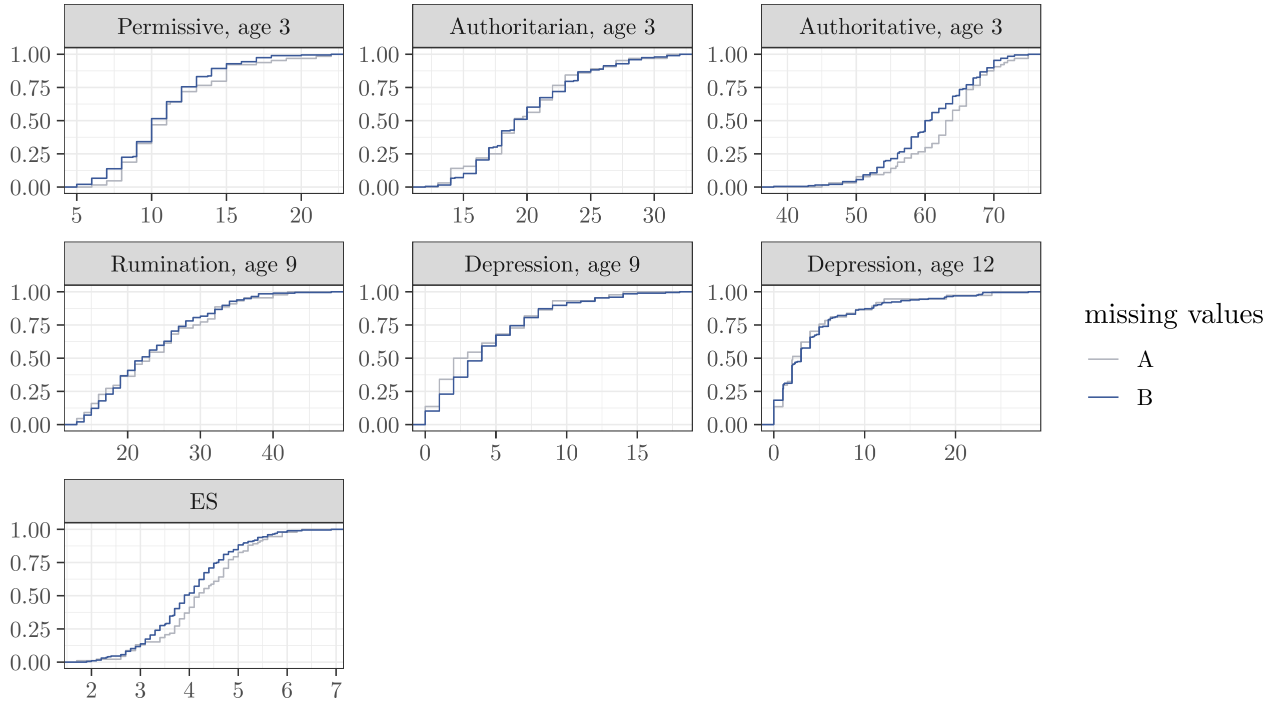

Figure 3: Empirical cumulative distributions of model variables depending on the presence of missing data. A refers to subjects with missing data, B to the sample used in the paper ($n = 196$).

2 Posterior distributions

In Figure 1 is depicted the complete target model of the current paper, tested for permissive parenting, authoritarian parenting and authoritative parenting.

Each model was fitted using the Bayesian MCMC estimation method implemented in the STAN probabilistic programming language (Carpenter et al., 2017; Stan-Development-Team, 2018) coupled with R-packages `blavaan` (Merkle & Rosseel, 2018) and `rstan` (Stan Development Team, 2020); for each model we sampled the posterior distributions of parameters by running MCMC chains with at least 4000 replicates each. We considered the interval $[-0.1, 0.1]$ as the set of null values, in other words we considered parameter values falling in this interval representing a substantially null effect (Region of Practical Equivalence – ROPE; Kruschke, 2018).

Figures 4, 5 and 6 represent posterior distributions of model parameters obtained on the sample described in the paper ($n = 196$ subjects) for permissive, authoritarian and authoritative Parenting style repsectively. Each posterior is based on 12000 effective replicates. Dashed vertical lines indicate the 90% Highest Posterior Density Interval (HPDI, i.e. the interval containing the 90% of posterior values) and green area represents the proportion of HPDI not included in the ROPE.

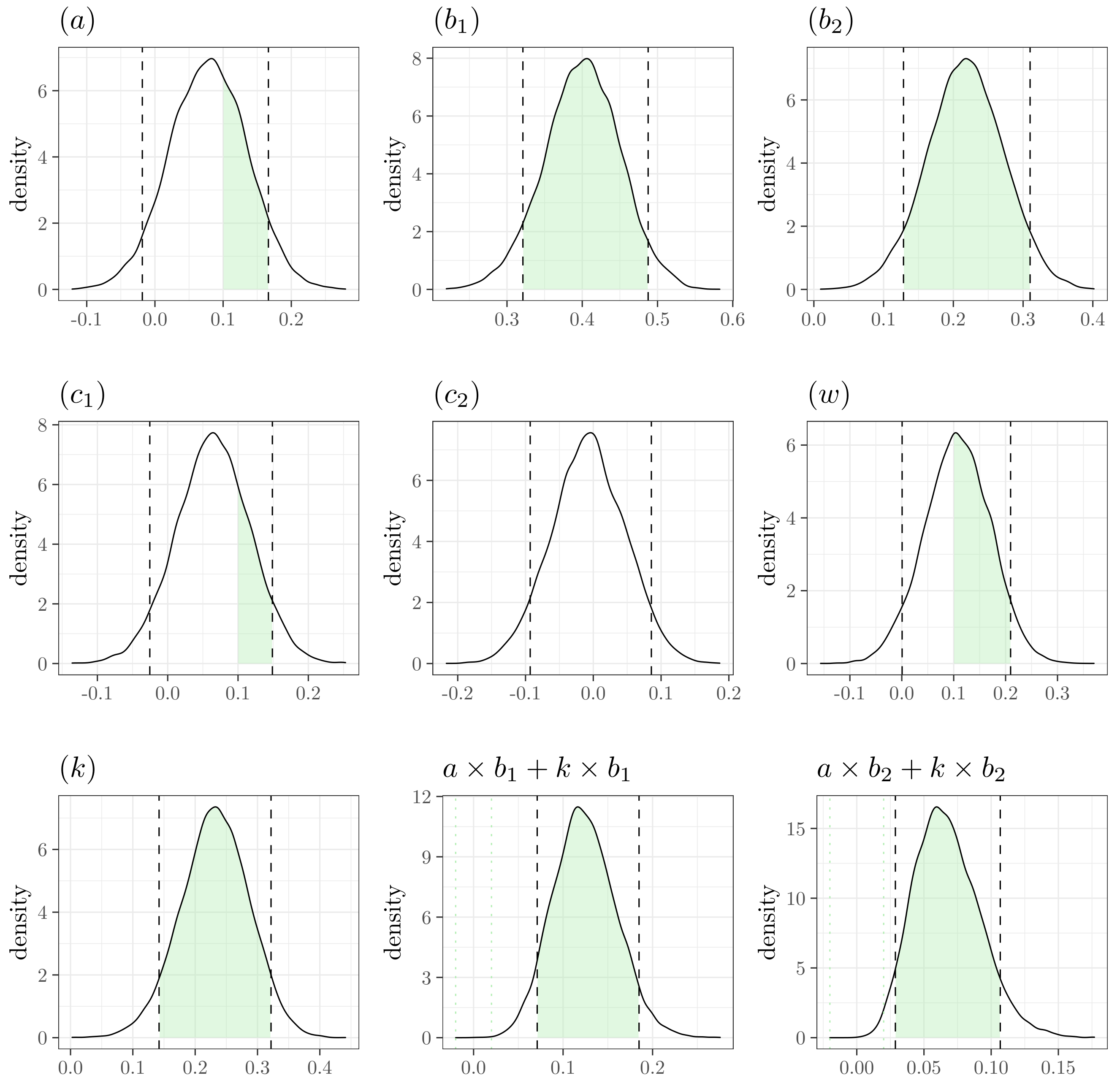

Figure 4: Permissive parenting. Posterior distributions of model parameters based on 12000 replicates. Dashed vertical lines indicate the 90% HPDI and green area represents the proportion of HPDI not included in the ROPE.

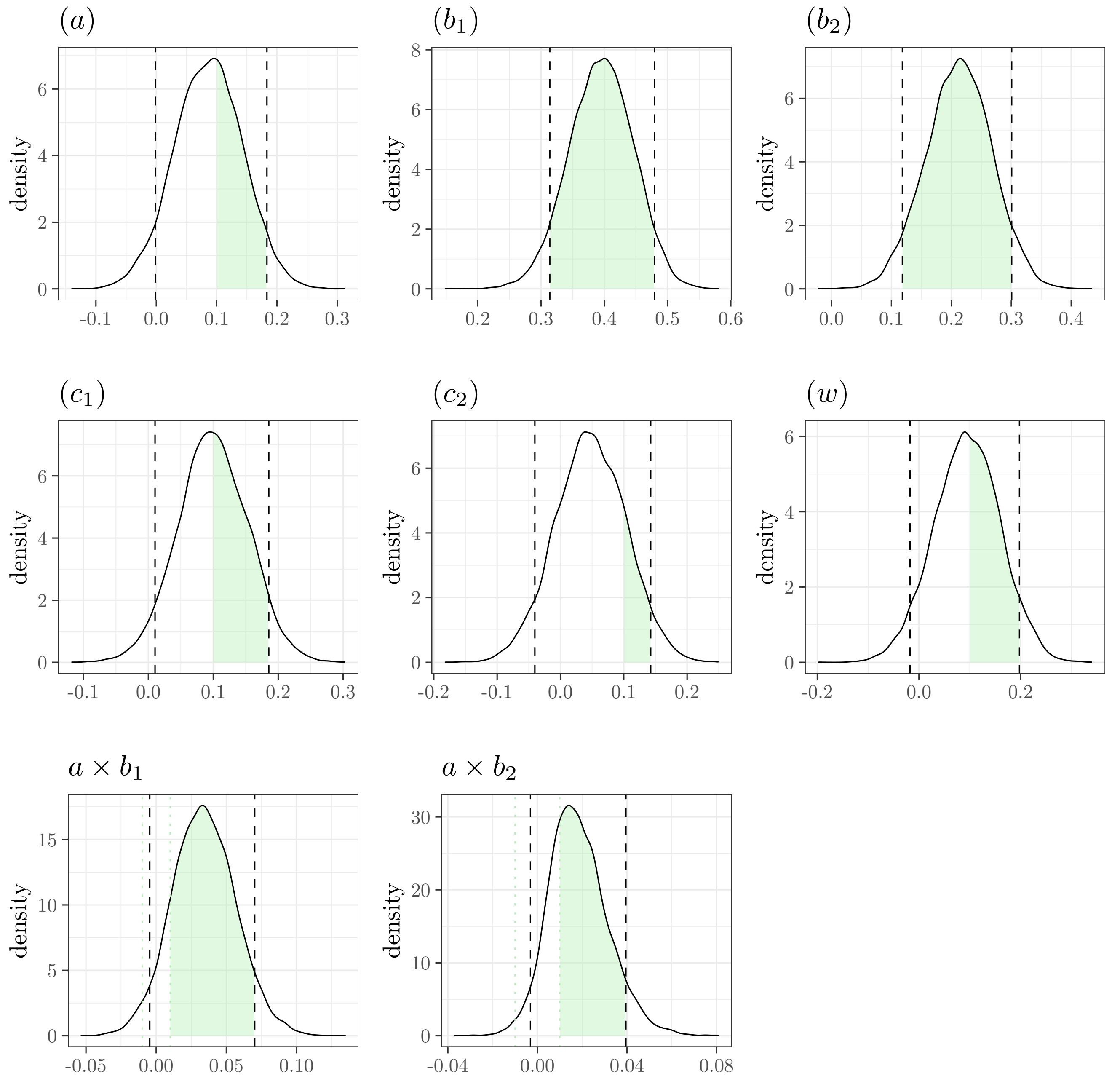

Figure 5: Authoritarian parenting. Posterior distributions of model parameters based on 12000 replicates. Dashed vertical lines indicate the 90% HPDI and green area represents the proportion of HPDI not included in the ROPE.

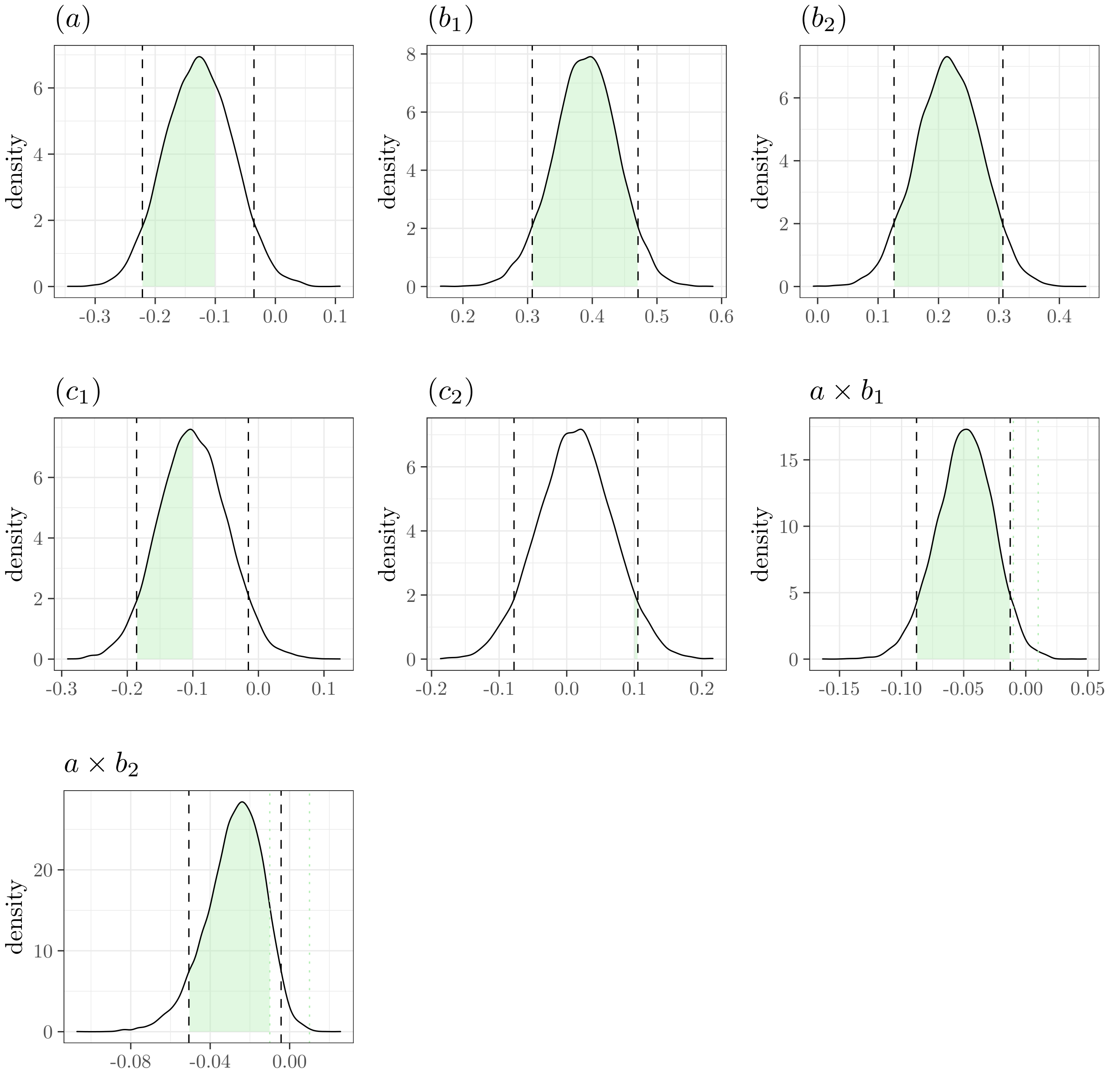

Figure 6: Authoritative parenting. Posterior distributions of model parameters based on 12000 replicates. Dashed vertical lines indicate the 90% HPDI and green area represents the proportion of HPDI not included in the ROPE.

3 Data imputation and Sensitivity analysis

In the paper only subjects for which responses were available at both age 9 and 12 were considered for the analyses. Since for 18 subjects data were not available at age 12, we replicated the analyses by imputing data. Results obtained with the imputation were overall comparable to results obtained with no imputed data. Hence, in the paper we reported results with no imputed data. Details of the analyses are reported below.

We performed a Multiple Imputation through Bayesian Bootstrap Predictive Mean Matching (BBPMM; Meinfelder & Schnapp, 2015). This allowed us to refit models on a sample of 214 subjects, instead of 196. In order to evaluate the impact of the imputation on estimated parameters, we performed a sensitivity analysis. More specifically, we replicated the imputation for 25 times, and we repeated for each replicate the estimation of model parameters adopting the same informative priors described in the paper (see the analytic plan section).

Each model was fitted using the Bayesian MCMC estimation method implemented in the STAN probabilistic programming language (Carpenter et al., 2017; Stan-Development-Team, 2018) coupled with R-packages `blavaan` (Merkle & Rosseel, 2018) and `rstan` (Stan Development Team, 2020); each posterior is based on 12000 effective replicates.

The Figures 7, 8 and 9 represent posterior distributions of model parameters obtained on the actual sample of 196 subjects (black lines) compared to the posteriors obtained with imputed data ($n = 214$, gray lines). Vertical dashed lines indicate the 90% HPDI, green area represents the proportion of HPDI not included in the ROPE both referred to the sample of 196 subjects.

3.1 Model with Permissive parenting

All parameters estimation were stable and comparable to parameters obtained on the actual sample (see Fig. 7). Clearly, parameters directly related to the variable with imputed data (i.e., Depression, age 12) were those showing the largest variability compared to results obtained without imputation. However, this variability did not change substantially the estimate (i.e. the mean of posterior distribution) of parameter b_2 – effect of Rumination, age 9 on Depression, age 12 – that appeared stable around the original estimate (0.22). For parameter c_2 – effect of Parenting, age 3 on Depression, age 12 – the increase of the estimate was negligible and from -0.01, obtained without data imputation, to an average estimate of about 0.01, obtained with data imputation. For parameter c_1 – effect of Parenting, age 3 on Depression, age 9 we observed a small increase in estimated posterior mean – from 0.06 (without imputation) to 0.08 (with imputation). For all other parameters no difference between posterior distributions obtained with and without imputation were identified.

3.2 Model with Authoritarian Parenting

Figure 8 refers to authoritarian parenting best model, i.e. the model without the interaction effect between parenting, age 3 and Environmental Sensitivity, age 3 on rumination, age 9. As it was for the model with permissive parenting, parameters directly related to the variable with imputed data (b_2 and c_2) showed the largest variability in posterior distributions in respect to parameters obtained without imputing data. This variability did not change substantially neither the estimate of parameter b_2, nor of parameter c_2. Note that there is only a single case in which the imputation produced a posterior that differed from the others and that was close to zero. Parameter c_1 showed a small increase of estimates from 0.1 (without imputation) to 0.12 (with imputation).

3.3 Model with Authoritative Parenting

Figure 9 refers to authoritative parenting best model, i.e. the model not including the variable Environmental Sensitivity, age 3. Again, in this model parameters directly related to the variable with imputed data (b_2 and c_2) showed the largest variability in posterior distributions compared to that obtained without imputing data. This variability did not change substantially the estimate neither of parameter b_2 nor of parameter c_2. Parameter c_1 showed a small increase from -0.1 (without imputation) to -0.13 (with imputation).

Summarizing, results with and without imputed data were overall stable. Hence, we reported in the paper results based on subjects for which actual data were available.

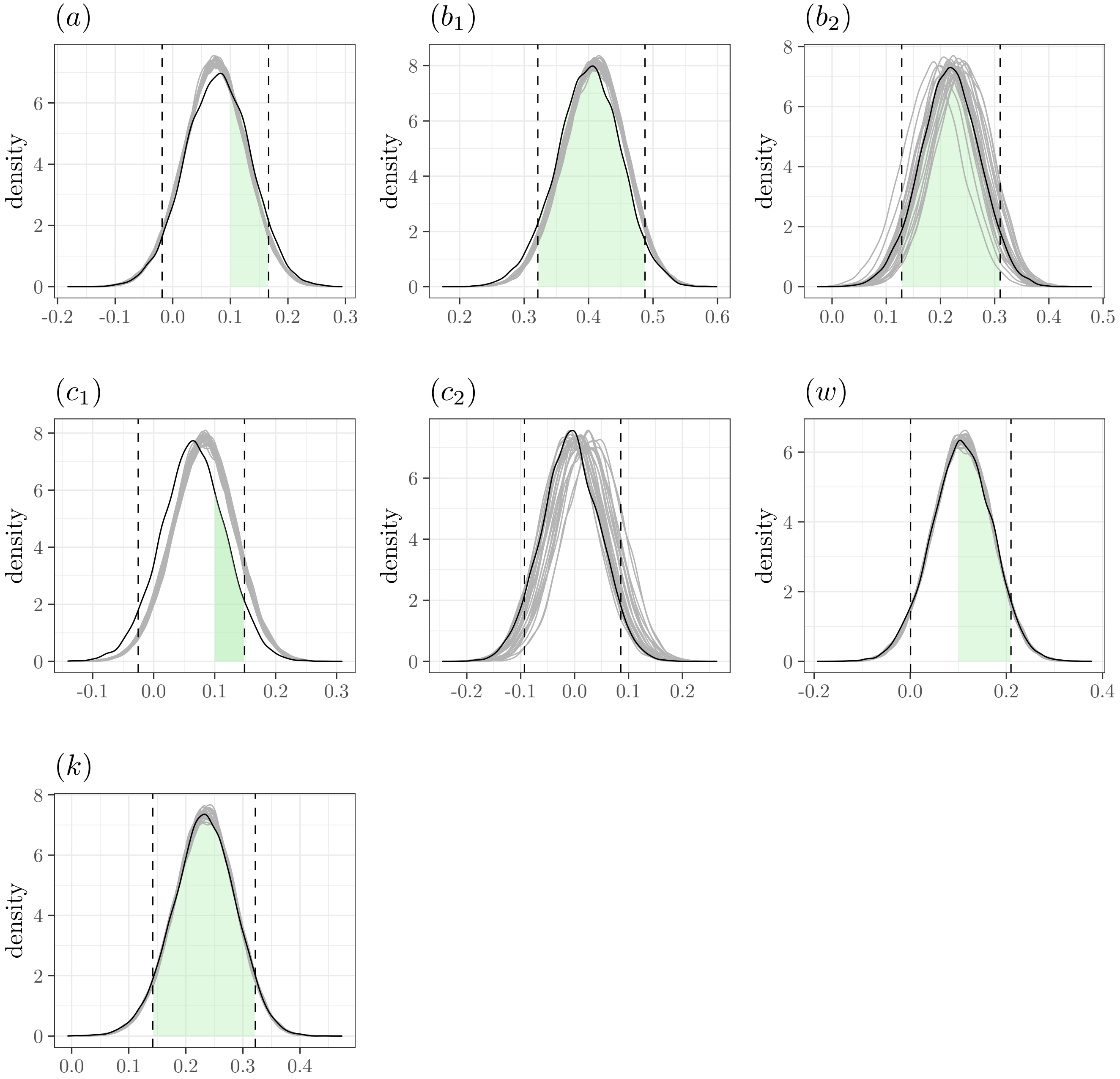

Figure 7: Permissive parenting. Posterior distributions of model parameters based on 12000 replicates. Black lines are the posteriors obtained in the sample without data imputation ($n = 196$), gray lines are the posteriors obtained in the sample with imputated data ($n = 214$). Dashed vertical lines indicate the 90% HPDI and green area represents the proportion of HPDI not included in the ROPE, both referred to sample without imputed data($n = 196$).

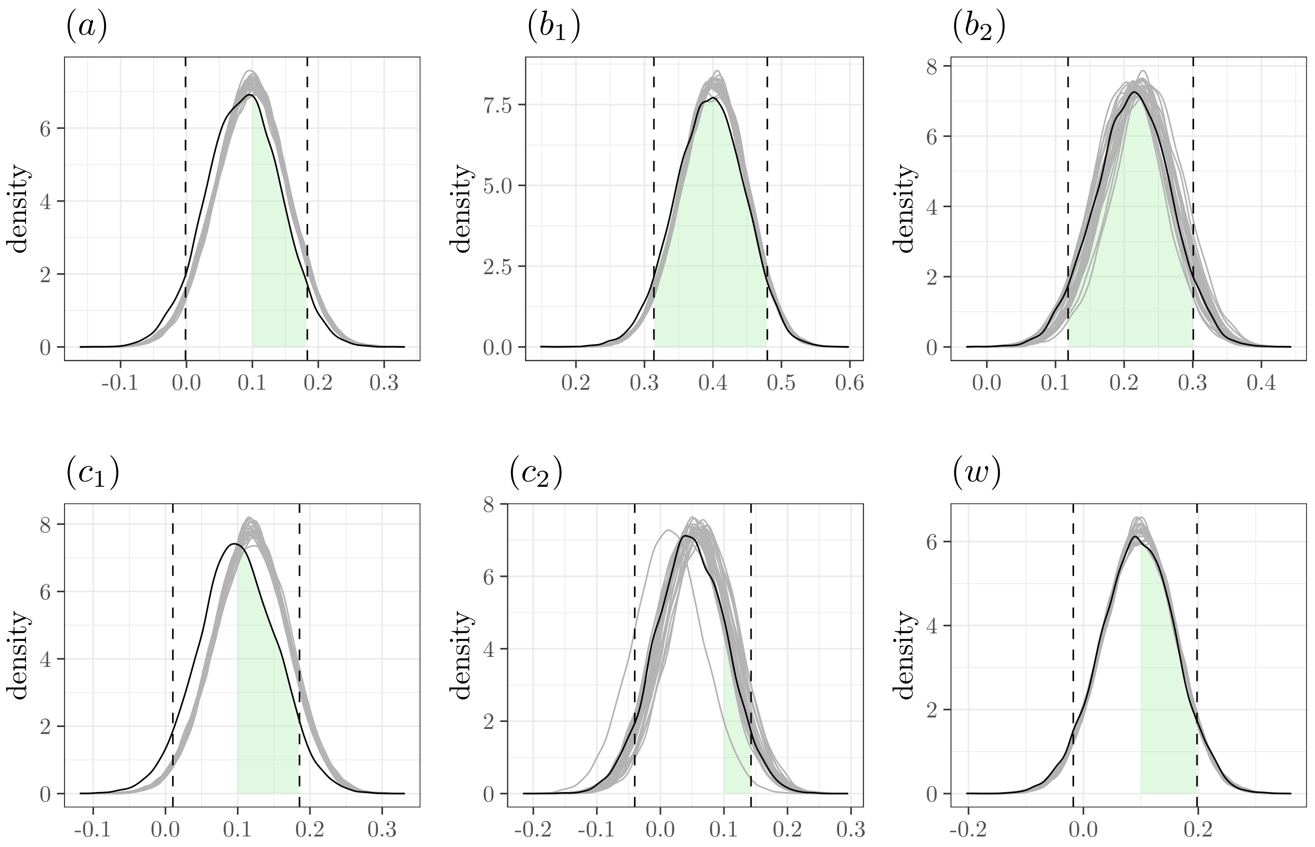

Figure 8: Authoritarian parenting. Posterior distributions of model parameters based on 12000 replicates. Black lines are the posteriors obtained in the sample without data imputation ($n = 196$), gray lines are the posteriors obtained in the sample with data imputation ($n = 214$). Dashed vertical lines indicate the 90% HPDI and green areas represent the proportion of HPDI not included in the ROPE, both referred to sample without data imputation ($n = 196$).

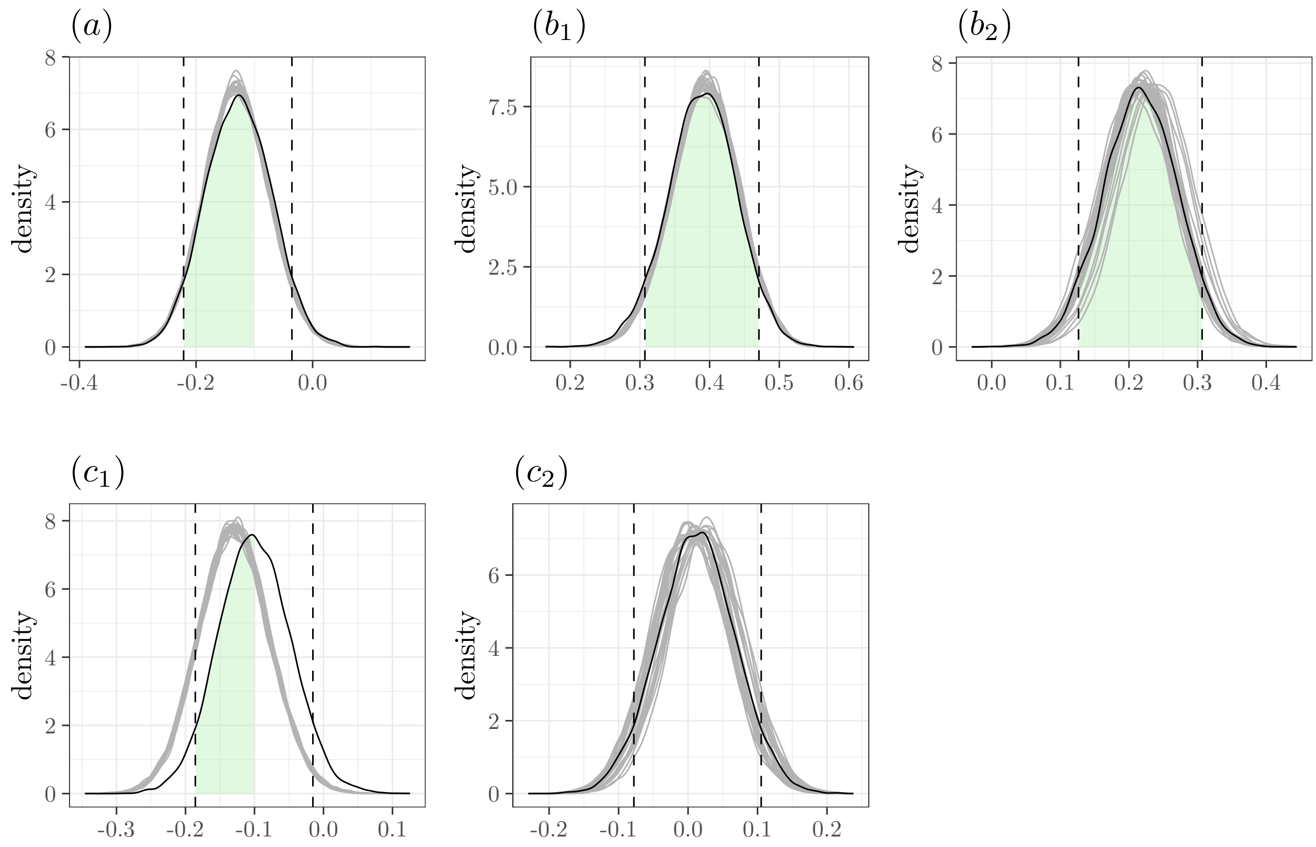

Figure 9: Authoritative parenting. Posterior distributions of model parameters based on 12000 replicates. Black lines are the posteriors obtained in the sample without data imputation ($n = 196$), gray lines are the posteriors obtained in the sample with data imputation ($n = 214$). Dashed vertical lines indicate the 90% HPDI and green areas represent the proportion of HPDI not included in the ROPE, both referred to sample without data imputation ($n = 196$).

4 Maximum likelihood estimates

As detailed in the introduction section and method section of the paper, we believe that the Bayesian models provided in the paper are more appropriate for the present data than their Frequentist counterparts. However, we also acknowledge that not all readers might be familiar with the chosen analytic approach. Hence, in this supplementary material section, we provide findings using more traditional techniques. Below, are reported AIC and Akaike weights related to the model comparison approach (tables 2, 3, 4), and Maximum Likelihood estimates for the best model (boxes 1, 2, 3) for each parenting style. Substantive conclusions are in all cases consistent with those derived from the models provided in text.

4.1 Permissive parenting

	AIC	w
Model 0	3363.36	0.00
Model 1	1633.54	0.10
Model 2	1633.51	0.10
Model 3	1629.26	0.81

Table 2: Permissive parenting: comparison of multivariate models. AIC = Akaike Information Criterion, w = Akaike weight.

```
lavaan 0.6-8 ended normally after 14 iterations

  Estimator                                         ML
  Optimization method                           NLMINB
  Number of model parameters                        14

  Number of observations                           196

Parameter Estimates:

  Standard errors                             Standard

Regressions:
                   Estimate  Std.Err  z-value  P(>|z|)   Std.lv  Std.all
  Dep_age9 ~
    Par_age3  (c1)    0.050    0.069    0.728    0.467    0.050    0.049
    Rum_age9  (b1)    0.355    0.068    5.226    0.000    0.355    0.350
  Dep_age12 ~
    Par_age3  (c2)   -0.038    0.073   -0.523    0.601   -0.038   -0.037
    Rum_age9  (b2)    0.146    0.072    2.020    0.043    0.146    0.143
  Rum_age9 ~
    Par_age3   (a)    0.073    0.070    1.038    0.299    0.073    0.072
    ES_age3    (w)    0.121    0.070    1.728    0.084    0.121    0.121
    Par_ES     (k)    0.187    0.064    2.903    0.004    0.187    0.202
```

Box 1: Permissive parenting (Model 3); maximum likelihood estimates. Note: `Dep_age9` = Depression at age 9; `Dep_age12` = Depression at age 12; `Rum_age9` = Rumination, age 9; `Par_age3` = Permissive parenting, age 3; `ES_age3` = Environmental Sensitivity, age 3; `Par_ES` = Permissive parenting × Sensitivity.

4.2 Authoritarian Parenting

	AIC	w
Model 0	3324.35	0.00
Model 1	1631.69	0.43
Model 2	1631.39	0.50
Model 3	1635.38	0.07

Table 3: Authoritarian parenting: comparison of multivariate models. AIC = Akaike Information Criterion, w = Akaike weight.

```
lavaan 0.6-8 ended normally after 16 iterations

  Estimator                                         ML
  Optimization method                           NLMINB
  Number of model parameters                        13
  Number of equality constraints                     1

  Number of observations                           196

Parameter Estimates:

  Standard errors                             Standard

Regressions:
                   Estimate  Std.Err  z-value  P(>|z|)   Std.lv  Std.all
  Dep_age9 ~
    Par_age3  (c1)    0.112    0.067    1.677    0.094    0.112    0.112
    Rum_age9  (b1)    0.349    0.067    5.180    0.000    0.349    0.345
  Dep_age12 ~
    Par_age3  (c2)    0.057    0.071    0.797    0.425    0.057    0.056
    Rum_age9  (b2)    0.139    0.072    1.924    0.054    0.139    0.136
  Rum_age9 ~
    Par_age3   (a)    0.082    0.070    1.171    0.241    0.082    0.083
    ES_age3    (w)    0.108    0.071    1.519    0.129    0.108    0.108
    Par_ES            0.000                               0.000    0.000
```

Box 2: Authoritarian parenting (Model 2); maximum likelihood estimates. Note: `Dep_age9` = Depression at age 9; `Dep_age12` = Depression at age 12; `Rum_age9` = Rumination, age 9; `Par_age3` = Authoritarian parenting, age 3; `ES_age3` = Environmental Sensitivity, age 3; `Par_ES` = Authoritarian parenting × Sensitivity.

4.3 Authoritative Parenting

	AIC	w
Model 0	3349.52	0.00
Model 1	1630.68	0.49
Model 2	1630.98	0.42
Model 3	1633.88	0.10

Table 4: Authoritative parenting: comparison of multivariate models. AIC = Akaike Information Criterion, w = Akaike weight.

```
lavaan 0.6-8 ended normally after 16 iterations

  Estimator                                         ML
  Optimization method                           NLMINB
  Number of model parameters                        12
  Number of equality constraints                     1

  Number of observations                           196

Parameter Estimates:

  Standard errors                             Standard

Regressions:
                   Estimate  Std.Err  z-value  P(>|z|)   Std.lv  Std.all
  Dep_age9 ~
    Par_age3  (c1)   -0.107    0.067   -1.588    0.112   -0.107   -0.107
    Rum_age9  (b1)    0.338    0.067    5.036    0.000    0.338    0.338
  Dep_age12 ~
    Par_age3  (c2)    0.032    0.071    0.450    0.652    0.032    0.032
    Rum_age9  (b2)    0.145    0.071    2.032    0.042    0.145    0.145
  Rum_age9 ~
    Par_age3   (a)   -0.143    0.071   -2.024    0.043   -0.143   -0.143
    ES_age3           0.000                               0.000    0.000
    Par_ES            0.000                               0.000    0.000
```

Box 3: Authoritative parenting (Model 1); maximum likelihood estimates. Note: `Dep_age9` = Depression at age 9; `Dep_age12` = Depression at age 12; `Rum_age9` = Rumination, age 9; `Par_age3` = Authoritative parenting, age 3; `ES_age3` = Environmental Sensitivity, age 3; `Par_ES` = Authoritative parenting × Sensitivity.

If you use Bayesian methods as another way
to compute p-values and confidence intervals,
you are missing the point.
Bayesian methods don't do the same things better;
they do different things, which are better.
(Allen Downey)

References

Carpenter, B., Gelman, A., Hoffman, M., Lee, D., Goodrich, B., Betancourt, M., ... Riddell, A. (2017). Stan: A probabilistic programming language. *Journal of Statistical Software*, *76*(1).

Kruschke, J. K. (2018). Rejecting or accepting parameter values in bayesian estimation. *Advances in Methods and Practices in Psychological Science*, *1*(2), 270–280.

Lionetti, F., Aron, E. N., Aron, A., Klein, D. N., & Pluess, M. (2019). Observer-rated environmental sensitivity moderates children's response to parenting quality in early childhood. *Developmental psychology*.

Meinfelder, F., & Schnapp, T. (2015). BaBooN: Bayesian Bootstrap Predictive Mean Matching - Multiple and Single Imputation for Discrete Data [Computer software manual]. Retrieved from `https://CRAN.R-project.org/package=BaBooN` (R package version 0.2-0)

Merkle, E. C., & Rosseel, Y. (2018). blavaan: Bayesian Structural Equation Models via Parameter Expansion. *Journal of Statistical Software*, *85*(4), 1–30. doi: 10.18637/jss.v085.i04

Pastore, M., & Calcagnì, A. (2019). Measuring distribution similarities between samples: A distribution-free overlapping index. *Frontiers in Psychology*, *10*, 1089. Retrieved from `https://doi.org/10.3389/fpsyg.2019.01089`

Stan Development Team. (2020). *RStan: the R interface to Stan.* Retrieved from `http://mc-stan.org/` (R package version 2.21.2)

Stan-Development-Team. (2018). Stan modeling language user's guide and reference manual, version 2.18.0 [Computer software manual]. Retrieved from `http://mc-stan.org`
